# An Immersive P300 Brain–Computer Interface Based on 3D Morphological Stimuli and Self-Adaptive Bayesian Linear Discriminant Analysis

**DOI:** 10.3390/biomimetics11060381

**Published:** 2026-06-01

**Authors:** Junhong Luo, Mengnan Zhu, Yongbo Xiao, Yuanhao Long, Xiaoting Zhang, Hui Cao, Javid Atai, Jing Xiao, Xuesong Chen

**Affiliations:** 1 School of Artificial Intelligence, Guangzhou Maritime University, Guangzhou 510725, China; luojunhong1@mails.gdut.edu.cn (J.L.); mengnanzhu1013@163.com (M.Z.); longyuanhao@gzmtu.edu.cn (Y.L.); zhangxt@e.gzhu.edu.cn (X.Z.); caohui@gzmtu.edu.cn (H.C.); 2 School of Mathematics and Statistics, Guangdong University of Technology, Guangzhou 510520, China; 3 School of Architecture and Art Design, Lanzhou University of Technology, Lanzhou 730050, China; liugang@lut.edu.cn; 4 School of Electrical and Computer Engineering, University of Sydney, Sydney, NSW 2006, Australia; javid.atai@sydney.edu.au

**Keywords:** brain–computer interface (BCI), P300, three-dimensional (3D), virtual reality (VR), adaptive stopping strategy

## Abstract

Conventional P300-based brain–computer interfaces (BCIs) commonly rely on two-dimensional (2D) visual flashing, which may induce visual fatigue and limit immersion, thereby restricting long-term usability and system performance. To address these limitations, this study proposes an immersive P300-BCI framework integrating a three-dimensional morphological stimulation paradigm, termed 3D-Morph, with self-adaptive Bayesian linear discriminant analysis (SA-BLDA). Instead of using color or luminance flickering, the proposed paradigm employs dynamic 2D-to-3D morphological transformations of virtual objects in a virtual reality environment to enhance target-related event-related potentials while preserving visual immersion. SA-BLDA further adjusts the number of stimulation rounds according to classification confidence to balance accuracy and interaction efficiency. Experiments with 24 participants showed that the proposed system outperformed the conventional 2D paradigm. In offline analysis, the proposed method achieved an average classification accuracy of 94.17% and an information transfer rate (ITR) of 25.50 bits/min, significantly outperforming the 2D paradigm (87.29% accuracy, 22.75 bits/min ITR, both p<0.001, Cohen’s d≥1.22). In online experiments, the 3D-Morph paradigm achieved an average accuracy of 91.46% and an ITR of 37.23 bits/min, compared with 83.96% and 28.74 bits/min for the conventional 2D paradigm (both p<0.01, Cohen’s d≥1.14). The average response time was reduced by 0.46 s (p<0.01, Cohen’s d=0.78), and the processing time per stimulation round (PT) of SA-BLDA was significantly reduced from 48.54±10.47 ms in the 2D paradigm to 26.40±9.41 ms in the 3D-Morph paradigm (p<0.01, Cohen’s d=2.34), corresponding to a 45.61% reduction in computational time per round. NASA-TLX evaluations indicated a significantly lower subjective workload across all dimensions (all p<0.05, Cohen’s d≥0.76). These results demonstrate that combining 3D-Morph stimulation with SA-BLDA can significantly improve classification performance, interaction efficiency, and user experience, providing a feasible framework for immersive and practical P300-BCI applications.

## 1. Introduction

Brain–computer interfaces (BCIs) provide a direct communication pathway between the human brain and external devices by bypassing peripheral nerves and muscles, thereby enabling novel forms of human–machine interaction [1,2,3]. Among various BCI paradigms, non-invasive electroencephalography (EEG)-based BCIs have attracted extensive attention due to their safety, low cost, and ease of deployment [4,5,6]. Common EEG-based BCI paradigms include steady-state visual evoked potentials (SSVEP), motor imagery (MI), and event-related potentials (ERPs). In particular, ERP-based BCIs have been widely adopted in attention-modulated interaction systems [7,8]. The P300 component, characterized by a positive deflection occurring approximately 300 ms after the presentation of a target stimulus, has been extensively applied in a wide range of BCI applications, such as spelling systems [9], robotic arm control [10,11], smart home and appliance control [12,13,14], and wheelchair navigation [15,16]. Nevertheless, most existing P300-BCI systems still adopt 2D matrix-based or symbolic visual interfaces. Although these interfaces are effective in laboratory settings, they may not sufficiently represent real-world interaction scenarios, leading to limited immersion, reduced ecological validity, and potential visual fatigue during long-term use [17].

In recent years, substantial research efforts have been devoted to improving the classification accuracy (ACC) of P300-based BCI systems, with a particular focus on the design of visual stimulation paradigms [18,19]. For example, Jin et al. combined P300 potentials with motion-onset visual evoked potentials (M-VEPs), achieving significantly higher online ACC than single-paradigm approaches (96% vs. 88%) [20]. Li et al. proposed single-character and region-based speller paradigms and demonstrated that region-based stimulation can effectively enhance P300 responses [9]. In addition, Speier et al. replaced conventional flashing symbols with familiar face stimuli, resulting in an increase in online ACC from 85.9% to 94.21% [21]. Despite these advances, most existing studies are still based on two-dimensional (2D) visual stimulation interfaces. Previous research has shown that prolonged exposure to repetitive 2D flashing stimuli may induce visual fatigue and reduce user subjective engagement, which in turn degrades long-term system performance and usability [22].

Compared with conventional two-dimensional (2D) paradigms, P300-based BCIs implemented in realistic three-dimensional (3D) virtual environments are capable of providing a more immersive user experience and, in many cases, achieving superior system performance [23,24]. For instance, Qu et al. introduced a 3D P300 speller in which stereoscopic virtual buttons replaced traditional 2D elements, and their online experiments demonstrated a higher average ACC for the 3D interface (94.0%) compared to the 2D counterpart (89.1%) [25]. Similarly, Korkmaz et al. proposed a P300 stimulation paradigm based on 3D animated flashing and reported an improvement in ACC from 89% to 91% under identical stimulation conditions when compared with a conventional 2D row–column paradigm [26]. In addition, Niu et al. developed a hybrid SSVEP–3D system that deviates from standard SSVEP paradigms, showing that the average recognition ACC increased from 92.8% to 95.6% in a 3D virtual environment [27]. Collectively, these studies indicate that integrating BCI systems with 3D virtual scenes is not only technically feasible but also beneficial for enhancing interaction effectiveness and user subjective engagement [28].

Nevertheless, existing visual stimulation methods mainly rely on color or luminance switching, which may not effectively alleviate visual fatigue [29,30,31]. In contrast, using morphological changes of target objects as visual stimuli has been reported to reduce visual fatigue and enhance target-related ERP components [32]. For example, Li et al. proposed a green familiar faces (GFF) spelling paradigm for a P300 speller, in which conventional character flashing was replaced by alternating green familiar faces and characters. Their online results showed that the GFF paradigm elicited larger mean ERP amplitudes than the conventional spelling paradigm, and increased the average classification accuracy from 75.6% to 86.1% [33]. Compared with conventional screen-based interfaces, 3D environments provide embodied and more natural interaction contexts, which may improve user subjective engagement and further enhance ERP responses [34]. In this context, 3D virtual objects can be manipulated through morphological changes, such as scaling, deformation, or structural transformation, thereby providing more natural and spatially meaningful target cues. Such morphology-based stimulation may reduce visual discomfort and enhance attentional engagement [35]. However, most existing immersive BCI studies mainly focus on scene construction, feedback presentation, or task execution, whereas the role of stimulus morphology in eliciting reliable P300 responses remains insufficiently investigated.

On the other hand, conventional P300-based BCI systems typically employ a fixed number of stimulus rounds (e.g., 10 rounds or 15 rounds) to ensure reliable ACC [25,36]. However, such a design inevitably prolongs the response time (RT), thereby limiting the information transfer rate (ITR) [37]. To address this trade-off, recent studies have introduced adaptive or dynamic stopping strategies that aim to improve ITR without significantly compromising ACC [38,39,40]. For instance, Lenhardt et al. proposed a dual-threshold-based dynamic stopping approach, which reduced the fixed 10 rounds in the online phase to an average of 4 rounds, achieving an ACC of 87.5% and an ITR of 29.35 bits/min, both higher than those of the conventional fixed-round method (ACC: 80%, ITR: 25 bits/min) [41]. Vo et al. developed an integrated framework combining classification, dynamic stopping, and adaptive learning, reducing the average number of rounds to 7.49 while achieving an ACC of 89.44% and an ITR of 32.51 bits/min, compared with a conventional 15-round P300 speller [42]. More recently, Ahmadi et al. proposed a Bayesian dynamic stopping approach based on posterior probability thresholds, demonstrating that the ITR could be significantly improved without a statistically significant loss in ACC compared to fixed-round methods [43]. These findings collectively demonstrate that adaptive/dynamic stopping mechanisms can effectively overcome the limitations of fixed-round strategies by reducing redundant stimulus repetitions, thereby improving ITR while maintaining ACC [39,44].

Despite these advances, several limitations remain in current P300-BCI and immersive BCI studies. First, conventional P300-BCI systems still mainly rely on 2D matrix-based interfaces and color- or luminance-flashing stimuli, which may limit immersion and natural interaction. Second, although 3D virtual scenes have been increasingly introduced into BCI systems, the influence of 3D morphological stimulation on P300 elicitation and classification performance has not been systematically examined. Third, many P300-BCI systems still use fixed stimulation rounds or subject-dependent calibration strategies, making it difficult to simultaneously optimize classification accuracy, response time, and user workload in online interaction.

Motivated by these advances, this study proposes an immersive 3D morphology-based adaptive P300-BCI interaction system for virtual environments. The main contributions of this work are summarized as follows.

1. Realistic 3D virtual objects are constructed in a VR environment to replace conventional matrix-based 2D buttons, thereby enhancing visual immersion and user subjective engagement;
2. A novel stimulation paradigm based on 2D-to-3D morphological transformation (termed the 3D-Morph paradigm) is developed, in which P300 responses are elicited through object shape changes rather than color or luminance flickering, leading to enhanced target-related ERP components and reduced visual fatigue;
3. A self-adaptive classification strategy based on Bayesian linear discriminant analysis (BLDA), referred to as SA-BLDA, is proposed to dynamically adjust the number of stimulus rounds according to classification confidence, thereby achieving an effective balance between ACC and RT.

## 2. Methods

### 2.1. Subject

A total of 24 healthy, right-handed participants took part in this study, including 16 males and 8 females, aged 20–25 years. All participants had normal or corrected-to-normal vision and normal hearing, no color vision deficiency, and no self-reported history of neurological, psychiatric, or severe visual disorders. None of them reported taking medication that could affect cognitive function or EEG responses. Before the formal experiment, all participants completed a brief VR adaptability screening and task familiarization session to ensure that they could tolerate the immersive environment and fully understand the experimental procedure.

This study was approved by the Ethics Committee of Guangdong Work Injury Rehabilitation Hospital, China (protocol number: AF/SC-07/2024.53; approval date: 6 September 2024), and all participants provided written informed consent before participation. All procedures were conducted in accordance with the Declaration of Helsinki.

### 2.2. Signal Acquisition

**Figure 1:**
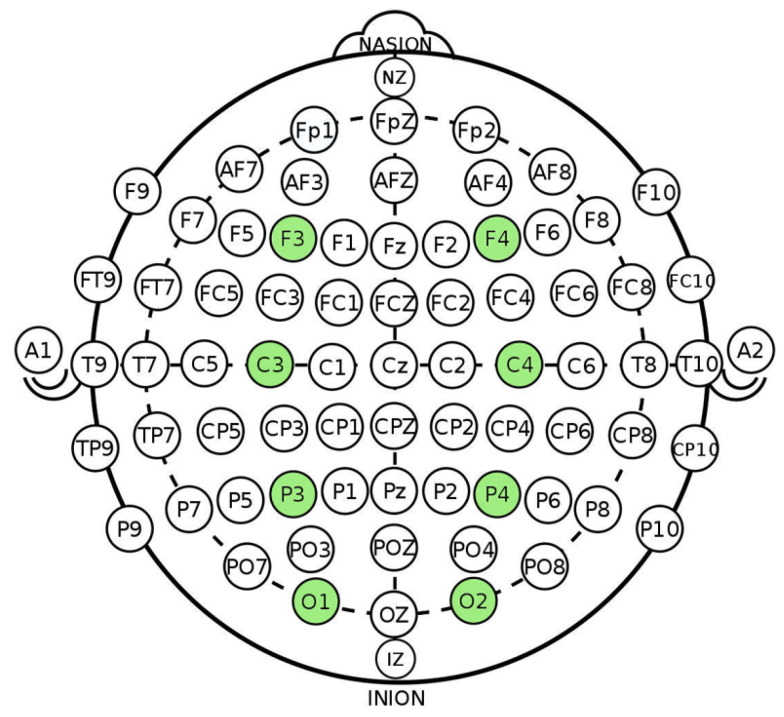
Scalp EG electrode placement based on the international 10–20 system.Green electrodes represent the selected channels used in this experiment.

EEG signals were acquired using a system developed by Brainnov Electronic Technology Co. (Qingdao, China), with a sampling rate of 250 Hz. Electrode placement followed the international 10–20 system, with Ag/AgCl electrodes positioned at Fz, Cz, P3, P4, P7, P8, O1, and O2, covering the frontocentral, parietal, posterior temporal, and occipital regions (Figure 1). The electrodes were integrated into an electrode cap and used with conductive gel to maintain electrode impedance below 10 kΩ.

### 2.3. System Design

**Figure 2:**
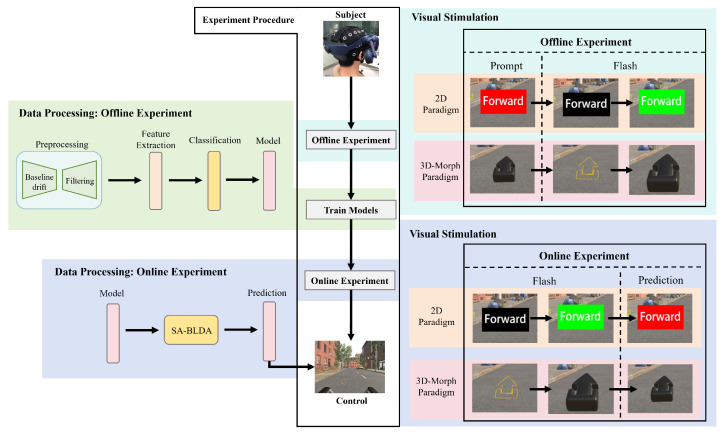
Architecture of the hybrid BCI system.

Figure 2 illustrates the proposed P300-based BCI system, which integrates EEG signal acquisition with virtual reality (VR) feedback. During the experiment, participants wore a Pico 4 Pro VR headset, and P300 ERPs were elicited through visual stimulation in the virtual environment.

**Figure 3:**
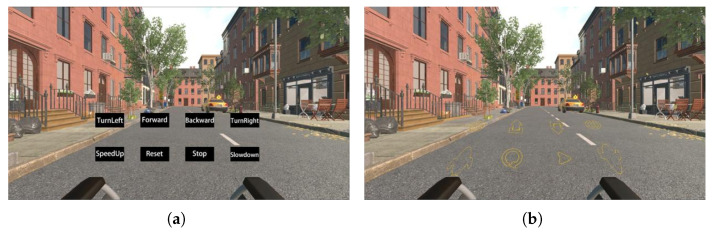
Stimulation interfaces of the 2D paradigm and 3D-Morph paradigm in the virtual environment. (**a**) Stimulation interface of the 2D paradigm. (**b**) Stimulation interface of the 3D-Morph paradigm.

In the virtual interface, the 2D planar stimulation interface (Figure 3a) was replaced by realistic 3D objects (Figure 3b) as stimulation targets, incorporating subtle morphological changes to preserve immersion. In the 2D paradigm, stimulation followed the conventional P300 speller design: buttons were black in the idle state, alternated between black and green during activation, and turned red to indicate feedback. In contrast, the 3D interface replaced color flashing with morphological transformation from a planar to a three-dimensional form during activation. Upon classification output, visual feedback was provided by enlarging the selected 3D object to 1.5 times its original size, thereby enhancing the perception of interaction outcomes.

### 2.4. Experiment Procedure

A within-subject crossover design was adopted in this study, in which each participant completed experiments under both the 3D-Morph paradigm and the conventional 2D paradigm. The two paradigms differed only in the visual stimulation mode of the interactive buttons, while all other experimental conditions, including task scenario, functional settings, and stimulation parameters, were kept identical to eliminate confounding factors.

The experimental scenario was developed using the Unity engine with a resolution of 1920 × 1080, featuring a realistic open-block virtual environment to support the virtual wheelchair control task. The stimulation interface consisted of eight functional buttons arranged in a 2 × 4 matrix, corresponding to the commands: Turn Left, Forward, Backward, Turn Right, Speed Up, Reset, Stop, and Slow Down.

Stimuli were presented in a pseudorandom sequence to ensure that two consecutive stimuli did not occur in the same row or column, thereby reducing spatial attention bias. A single flash of an individual button was defined as one epoch with a duration of 100 ms. A stimulation round consisted of one complete sequence in which all eight buttons were flashed once, with an inter-stimulus interval (ISI) of 120 ms between consecutive flashes. Each trial comprised ten consecutive stimulation rounds, and a rest interval of 1.5 s was provided between rounds to alleviate visual fatigue. These stimulation parameters (flash duration of 100 ms and ISI of 120 ms) were consistent with the classical P300 paradigm and ensured reliable elicitation of P300 event-related potentials.

All experiments were conducted on a workstation equipped with an RTX 4060 graphics card and 16 GB of RAM. During the experiments, system resource utilization remained stable at 62–68%, and GPU memory usage was approximately 1.8 GB, confirming that the hardware configuration met the real-time rendering requirements of the VR environment.

All participants completed both offline and online experiments under the two paradigms. Taking the offline experiment of the 3D-Morph paradigm as an example, the eight command buttons were represented by 3D objects with a physical size of 0.79 × 0.79 cm and a visual angle of 0.75°. Three seconds before the onset of each trial, the target object was enlarged to 1.5 times its original size as a cue, and participants were instructed to memorize its position. During stimulus presentation, participants performed a visual counting task by mentally counting the number of times the target button underwent morphological transformation, while passively fixating on non-target stimuli. A 3 s rest interval was provided after each trial before the next trial commenced.

For the offline experiment of the 2D paradigm, a conventional 4 × 2 matrix layout was adopted. During the cue phase, the target button was highlighted in red, and stimulation was delivered through black–green color flashing. The functional mapping, stimulation parameters, and number of trials were identical to those of the 3D-Morph paradigm to ensure consistency.

The online experiment aimed to evaluate real-time control performance under both paradigms. The self-adaptive Bayesian linear discriminant analysis (SA-BLDA) algorithm (see Section 2.5.2 for details) was employed to dynamically adjust the number of stimulation rounds per trial within a range of 3–7 rounds, thereby balancing classification accuracy and response speed. For the 3D-Morph paradigm, a total of 20 commands were randomly assigned. During a 3 s preparation phase, participants identified the target button, and during stimulation, they maintained fixation on the target while performing the visual counting task. When the classification confidence exceeded the predefined threshold for three consecutive rounds, the system generated a control output and provided visual feedback by enlarging the target object to 1.5 times its original size for 1 s, simultaneously executing the corresponding wheelchair command.

To minimize learning effects, an AB/BA crossover design was implemented. The 24 participants were randomly divided into two groups: Group A (n=12) completed the 3D-Morph paradigm first, followed by the 2D paradigm, whereas Group B (n=12) followed the opposite order. A 48 h interval was maintained between the two experimental sessions. All experiments were conducted under identical environmental conditions, and the VR rendering frame rate was maintained at 60 Hz to avoid potential confounding effects related to visual fatigue.

### 2.5. Classification Algorithm

#### 2.5.1. Bayesian Linear Discriminant Analysis (BLDA)

Bayesian Linear Discriminant Analysis (BLDA) was employed for P300 classification. Compared with conventional linear discriminant analysis (LDA), BLDA provides superior performance under small-sample or high-noise conditions [38,39].

Let $f\in R^{N}$ denote the input feature vector, where *N* is the feature dimension. The corresponding feature matrix is $F={\left[f_{1},f_{2},\ldots ,f_{M}\right]}^{T}$, where *M* represents the number of training samples. The regression target is *y*, where target and non-target stimuli are labeled as 0 and 1, respectively. The BLDA model is formulated as

(1) $$y=\omega^{T}f+\varphi$$

where *y* is the regression target, $\omega \in R^{N}$ is the weight vector and φ represents independent Gaussian noise.

To account for potential bias terms in EEG data, a constant bias term δ is introduced, extending the weight vector to N+1 dimensions: $\tilde{\omega }={\left[\omega^{T},\delta \right]}^{T},\tilde{\omega }\in R^{N+1}$, with the corresponding extended feature vector $\tilde{f}={\left[f^{T},\delta \right]}^{T}$. A zero-mean Gaussian prior is imposed on the extended weight vector, i.e., $\tilde{\omega }∼N\left(0,\Lambda^{-1}\right)$, where $\Lambda =\mathrm{diag}(\lambda_{1},\lambda_{2},\ldots ,\lambda_{N+1})$ is an (N+1)×(N+1) diagonal matrix whose diagonal elements $\lambda_{i}$ are set to small values to impose weak regularization.

Given the training set ${\left\{{\tilde{f}}_{i},y_{i}\right\}}_{i=1}^{M}$, the extended feature matrix $\tilde{F}={\left[{\tilde{f}}_{1},{\tilde{f}}_{2},\ldots ,{\tilde{f}}_{M}\right]}^{T}$ is used to train the model through an iterative procedure:

1. Eigenvalue decomposition is performed on $\tilde{F}$;
2. The model parameters are iteratively updated to optimize the prior inverse variance α and noise inverse variance β;
3. Iteration terminates when the changes in both α and β between consecutive iterations fall below a predefined convergence threshold or when the maximum number of iterations is reached.

After convergence, the log evidence is computed to evaluate model fitting. The optimal α, β, extended weight vector $\tilde{\omega }$, posterior precision matrix *P*, and posterior mean $u_{i}$ are retained as model parameters, yielding an individualized BLDA model for each participant.

In this study, the following parameters were used: δ=1, $\lambda_{i}=0.01$, initial prior inverse variance α=25, initial noise inverse variance β=1, convergence threshold $\varepsilon =10^{-4}$, and a maximum of 500 iterations.

#### 2.5.2. Self-Adaptive Bayesian Linear Discriminant Analysis (SA-BLDA)

**Figure 4:**
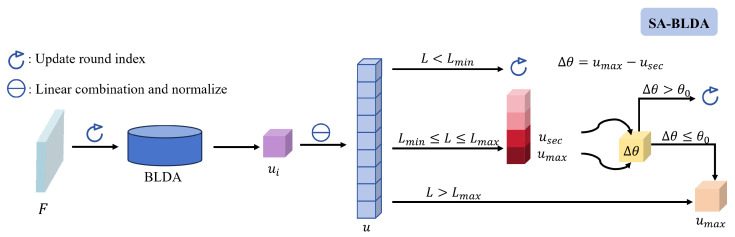
Flowchart of the SA-BLDA algorithm.

To improve real-time interaction efficiency while maintaining classification accuracy, a SA-BLDA algorithm was adopted. Unlike conventional fixed-round P300 classification strategies, SA-BLDA dynamically adjusts the number of stimulation rounds required for decision making based on classification confidence, thereby achieving a balance between accuracy and response speed. The structured pseudo-code of the detailed implementation is provided in Algorithm 1, and the conceptual workflow of SA-BLDA is illustrated in Figure 4. The definitions of the main parameters used in Algorithm 1 and Figure 4 are summarized in Table 1. By adaptively terminating stimulation once sufficient confidence is achieved, the SA-BLDA algorithm effectively reduces unnecessary stimulation rounds, shortens response time, and enhances the ITR, making it particularly suitable for real-time P300-based BCI applications.

To further evaluate the real-time feasibility of SA-BLDA, the processing time for each SA-BLDA decision round was calculated in our implementation. The online computation mainly consisted of feature updating, BLDA-based regression score calculation, comparison between the maximum and second-largest regression scores, and confidence-threshold judgment. These operations were completed within the online processing interval and did not introduce additional delay to the system operation. It should be noted that the absolute processing time of the SA-BLDA module may vary depending on the hardware configuration, EEG acquisition interface, software implementation, and VR rendering environment.
**Algorithm 1** Procedure of the SA-BLDA Algorithm**Require:** Minimum and maximum stimulation rounds $L_{\min}=4$, $L_{\max}=8$; decision threshold $\theta_{0}=0.2$**Ensure:** Predicted target $u_{\max}$  1:Initialize i=1, $L_{i}=1$, and Δθ=0  2:Obtain the initial feature vector $f_{i}$ and feature matrix $F_{i}$  3:**while** $L_{i}\leq L_{\max}$ **do**  4:    Update the averaged feature vector $f_{i}=\frac{1}{i}\sum_{m=1}^{i}f_{i,m}$ and feature matrix $F_{i}$  5:    **if** $L_{i}\geq L_{\min}$ **then**  6:        Apply BLDA to $F_{i}$ and obtain the regression score vector *u*  7:        Identify the maximum score $u_{\max}$ and the second-largest score $u_{\sec}$  8:        Compute the confidence difference $\Delta \theta =u_{\max}-u_{\sec}$  9:        **if** $\Delta \theta \geq \theta_{0}$ **or** $L_{i}=L_{\max}$ **then**10:           Output $u_{\max}$ as the predicted target11:           **break**12:        **end if**13:    **end if**14:    Update the stimulation round $L_{i+1}=L_{i}+1$15:    Set i=i+116:**end while**

### 2.6. Signal Processing

The EEG signal processing pipeline consisted of two connected stages: offline calibration and online recognition. To ensure consistency between the two stages, the same preprocessing and feature extraction procedures were applied, including baseline correction, band-pass filtering, downsampling, and feature normalization. During offline calibration, the extracted features were used to train an individualized BLDA model, which mapped EEG feature vectors to regression scores. During online recognition, real-time EEG epochs were processed using the identical pipeline and then fed into the offline-trained BLDA model. The resulting regression scores were further used by the SA-BLDA algorithm to adaptively determine the number of stimulation rounds and generate the target command in real time.

#### 2.6.1. Offline Signal Processing

**Figure 5:**
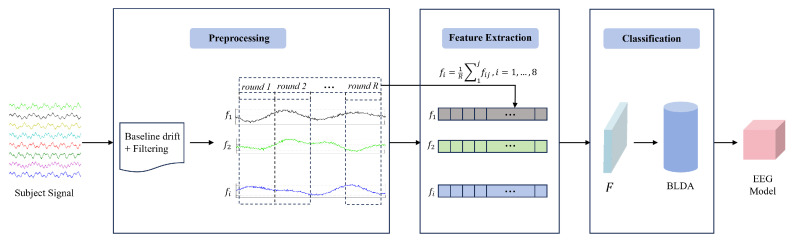
EEG signal processing pipeline in the offline experiment.

In the offline stage, EEG signals were processed to construct an individualized classification model (Figure 5). EEG preprocessing was first conducted to enhance signal quality. A 150 ms pre-stimulus interval was used for baseline correction, followed by band-pass filtering between 0.1 and 40 Hz using an infinite impulse response (IIR) filter. The filtered signals were then downsampled with a factor of 6 to reduce data dimensionality and computational load.

Subsequently, EEG epochs corresponding to the post-stimulus time window were extracted for feature construction. To suppress noise inherent in single-round EEG signals, feature vectors obtained from multiple stimulation rounds within each trial were averaged. Let $f_{i,j}$ denote the feature vector extracted from the *i*-th round of the *j*-th trial. The trial-level feature vector $f_{i}$ was computed as

(2) $$f_{i}=\frac{1}{R}\sum_{j=1}^{R}f_{i,j},$$

where *R* represents the number of stimulation rounds in a trial.

The feature vectors from all trials were then concatenated to form a feature matrix, which was normalized using L2-norm scaling to obtain the normalized feature matrix. This normalization step ensured comparable feature magnitudes across trials and improved classifier robustness.

Finally, the normalized feature matrix and corresponding labels were used to train the BLDA (see Section 2.5.1 for details) classifier, thereby completing the construction of an individualized offline EEG classification model.

#### 2.6.2. Online Signal Processing

For EEG signals acquired in real time during the online phase, the same preprocessing and feature extraction procedures as those used in the offline stage were applied, including baseline correction, band-pass filtering, downsampling, and feature normalization. Based on the extracted features, the SA-BLDA algorithm (see Section 2.5.2 for details) was employed to adaptively adjust the number of stimulation rounds and generate the target command in real time. This strategy maintains consistency with offline classification while improving real-time response speed.

In SA-BLDA, the initial control output threshold was set to $\theta_{0}=0.2$ based on pilot tests on calibration data and preliminary online runs. Several candidate thresholds (such as 0.1, 0.15, 0.2, 0.25, and 0.3) were compared by considering both classification accuracy and the average number of stimulation rounds. Lower thresholds increased the risk of premature incorrect outputs, whereas higher thresholds prolonged stimulation and reduced the information transfer rate. Therefore, $\theta_{0}=0.2$ was selected as a compromise between decision reliability and response speed. Moreover, $\theta_{0}$ was used as an initial confidence-control threshold rather than a fixed final decision boundary. The definitions and values of the main SA-BLDA parameters are summarized in Table 2.

### 2.7. Data Analysis Methods

#### 2.7.1. Performance Evaluation

Classification accuracy (ACC) is a fundamental performance metric in P300-based BCI systems, reflecting the proportion of correctly recognized commands among all issued commands. ACC is defined as

(3) $$ACC=\frac{T}{N},$$

where *T* denotes the number of correctly recognized commands and *N* represents the total number of issued commands.

In addition to classification accuracy, the information transfer rate (ITR) was calculated to evaluate the communication efficiency of the BCI system by jointly considering accuracy and response time, defined as the elapsed time from stimulus onset to the generation of a classification output. ITR is defined as

(4) $$ITR=60\left(\log_{2}N+ACC\log_{2}ACC+\left(1-ACC\right)\log_{2}\left[\frac{1-ACC}{N-1}\right]\right)/T,$$

where *T* is the target response time of the BCI system.

#### 2.7.2. Workload Evaluation

To assess subjective workload during interaction, the NASA Task Load Index (NASA-TLX) questionnaire was administered after each experimental condition. The NASA-TLX evaluates perceived workload across six dimensions: mental demand, physical demand, temporal demand, performance, effort, and frustration. Scores from these dimensions were combined to obtain an overall workload score for each participant and condition.

#### 2.7.3. Statistical Analyses

Given that all 24 participants completed both the 2D and 3D-Morph paradigm experiments, the data constituted paired observations. Accordingly, a two-tailed paired-samples *t*-test was employed to assess statistical significance for all performance metrics and task workload measures. Additionally, Cohen’s d was reported to assess the practical significance of the observed differences between conditions.

## 3. Results

### 3.1. Offline Experiment Results

**Figure 6:**
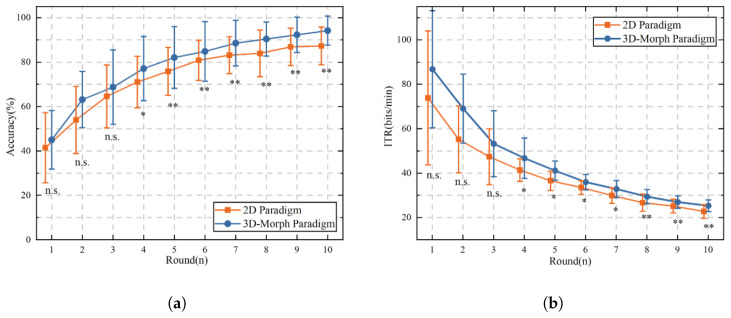
Average accuracy and average ITR results of 24 participants under different paradigms in the offline experiment. (**a**) Average accuracy of the 2D paradigm and the 3D-Morph paradigm. (**b**) Average ITR of the 2D paradigm and the 3D-Morph paradigm. Error bars indicate the standard error of the mean across participants. Statistical significance between the two paradigms is denoted as follows: * indicates (*p* < 0.05), ** indicates (*p* < 0.01), and n.s. indicates no significant difference.

Figure 6 illustrates the average system performance across 24 participants in the offline experiment. As shown in Figure 6a, the average classification accuracy (ACC) of both paradigms increases with the number of stimulation rounds. At the 10th round, the 3D-Morph paradigm achieves a higher average ACC (94.17%) compared to the conventional 2D paradigm (87.29%, p<0.001, Cohen’s d=1.30).

In contrast, the information transfer rate (ITR) decreases as the number of rounds increases (Figure 6b), due to the longer decision time. Nevertheless, the 3D-Morph paradigm consistently outperforms the 2D paradigm, achieving an average ITR of 25.50 bits/min at the 10th round, compared to 22.75 bits/min for the 2D paradigm (p<0.001, Cohen’s d=1.22). Furthermore, statistical analyses of ACC and ITR across different stimulation rounds are presented in Figure 6a and Figure 6b, respectively. For ACC (Figure 6a), no significant difference is observed between the two paradigms when the number of rounds is less than or equal to three (p>0.05, Cohen’s d=0.22), indicating that insufficient discriminative information is available at low stimulation rounds. Starting from the 4th round, a significant difference emerges (p≤0.05, Cohen’s d=0.35), and further increases to a highly significant difference from the 5th to the 10th round (p≤0.01, Cohen’s d=0.50), demonstrating that the 3D-Morph paradigm achieves superior classification performance under sufficient stimulus accumulation, with the largest effect size at the 10th round (Cohen’s d=1.30). For ITR (Figure 6b), no significant difference is observed at low stimulation rounds (round ≤ 3, p>0.05, Cohen’s d=0.35), and a significant difference emerges from rounds 4 to 7 (p<0.05, Cohen’s d=0.33). The significant difference persists from rounds 8 to 10 (p<0.01, Cohen’s d=0.60), indicating that the 3D-Morph paradigm maintains a stable advantage in ITR even with increased stimulation duration.

Overall, a significant improvement in ACC is observed from the 4th round onward, and the ITR shows a consistent significant advantage across all rounds with sufficient stimulus accumulation. These findings suggest that the 3D-Morph paradigm provides a more efficient trade-off between accuracy and speed, particularly in moderate-to-high-stimulation rounds.

**Figure 7:**
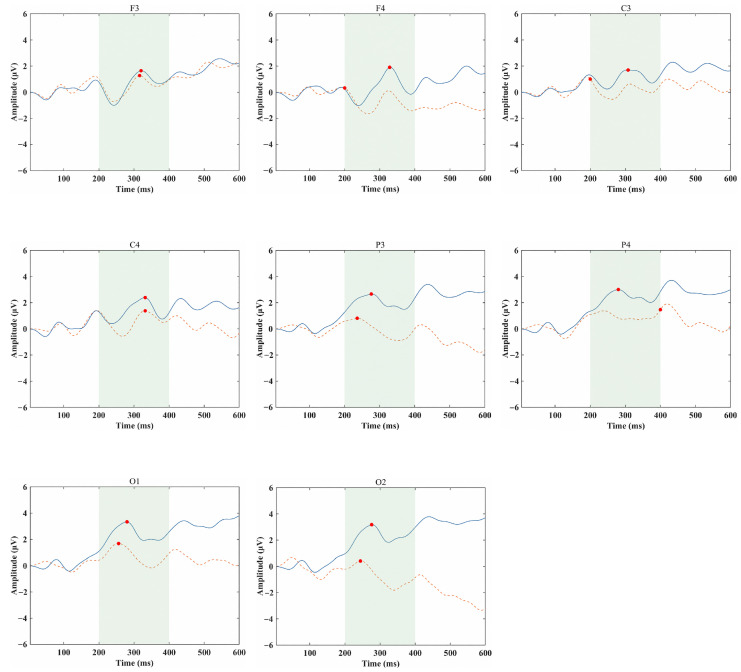
P300 peak amplitudes and latencies across eight EEG channels (F3, F4, C3, C4, P3, P4, O1, O2) for the 2D paradigm and 3D-Morph paradigm.

Figure 7 presents the P300 peak amplitude (μV) and peak latency (ms) within the canonical P300 time window (200–400 ms) across eight EEG channels (F3, F4, C3, C4, P3, P4, O1, and O2) for both the 2D and 3D-Morph paradigms, averaged over 24 participants. The results indicate that, under the 2D paradigm, P300 peak latencies are widely distributed across channels, ranging from 200 to 400 ms. Specifically, the earliest peaks are observed at the F4 and C3 channels (both at 200 ms), whereas the latest peak occurs at the P4 channel (400 ms). In contrast, the 3D-Morph paradigm exhibits a markedly more concentrated latency distribution, with all P300 peaks occurring within a narrower range of 276–328 ms. More specifically, the earliest peaks are observed at the P3 and O2 channels (both at 276 ms), while the latest peak appears at the F4 channel (328 ms). In addition, the P300 peak amplitudes elicited under the 3D-Morph paradigm are consistently higher than those under the 2D paradigm across all channels. This enhancement is particularly pronounced in posterior electrodes (P3, P4, O1, and O2), indicating that the proposed paradigm more effectively facilitates target-related neural responses in brain regions associated with visual attention and information processing.

### 3.2. Online Experiment Results

Table 3 and Table 4 summarize the individual online experimental results of 24 participants under the proposed 3D-Morph paradigm and the conventional 2D paradigm, respectively. Five performance metrics were evaluated, including the average number of stimulation rounds determined by the SA-BLDA algorithm, RT, ITR, ACC, and the processing time per stimulation round (PT). The results demonstrate that the 3D-Morph paradigm outperformed the 2D paradigm across all evaluation metrics. Specifically, under the adaptive stimulation control mode, the average number of stimulation rounds required for correct command recognition was 4.76 ± 0.37 for the 3D-Morph paradigm, which was significantly lower than that of the 2D paradigm (5.30 ± 0.64, p<0.01, Cohen’s d=0.95). Correspondingly, the average RT was reduced from 6.33 ± 0.52 s in the 2D paradigm to 5.87 ± 0.49 s in the 3D-Morph paradigm (p<0.01, Cohen’s d=0.78). Moreover, the PT of SA-BLDA was also significantly reduced under the 3D-Morph paradigm, decreasing from 48.54 ± 10.47 ms in the 2D paradigm to 26.40 ± 9.41 ms in the 3D-Morph paradigm (p<0.01, Cohen’s d=2.34), corresponding to a 45.61% reduction in PT.

In terms of classification performance, the 3D-Morph paradigm achieved an average classification accuracy of 91.46% ± 5.58%, which was significantly higher than that of the 2D paradigm (83.96% ± 5.47%, p<0.01, Cohen’s d=1.14). Notably, 23 out of the 24 participants attained classification accuracies that were equal to or higher than their corresponding performance under the 2D paradigm. Among them, six participants achieved classification accuracies of 95% or higher under the 3D-Morph paradigm, with the highest accuracy reaching 100%, whereas the maximum accuracy under the 2D paradigm was 95%. Furthermore, benefiting from enhanced ERP responses and the adaptive stimulation-round control strategy, the average ITR of the 3D-Morph paradigm reached 37.23 ± 5.20 bits/min, representing a 29.54% improvement over the 2D paradigm (28.74 ± 3.92 bits/min, p<0.01, Cohen’s d=1.35). Overall, compared with the conventional 2D flashing paradigm, the proposed 3D-Morph paradigm achieved superior online BCI performance in terms of ACC, RT, ITR, and PT under adaptive control conditions, with all metrics showing medium-to-very-large effect sizes.

### 3.3. Workload Evaluation Results

**Figure 8:**
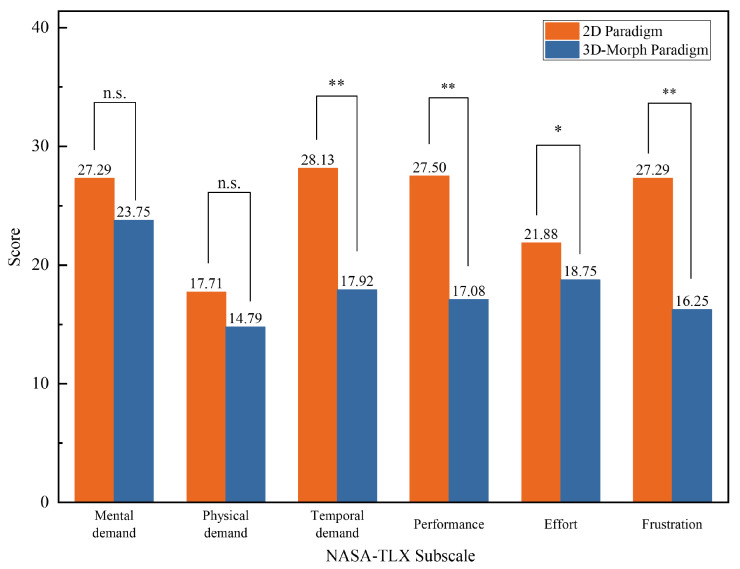
Workload evaluation results of the 2D paradigm and 3D-Morph paradigm based on the NASA-TLX questionnaire. “n.s.”, “*” and “**” denote the significance levels between the two paradigms, corresponding to p≥0.05, p<0.05 and p<0.01, respectively.

To quantitatively evaluate subjective workload differences between the two P300 stimulation paradigms, the NASA Task Load Index (NASA-TLX) was employed to assess 24 participants (S1–S24). After completing the online experiment, each participant independently rated the 2D and 3D-Morph paradigms across six dimensions (Figure 8).

The results show that the 3D-Morph paradigm yields lower average scores than the 2D paradigm across all six dimensions, including mental demand, physical demand, temporal demand, performance, effort, and frustration. Specifically, no significant differences are observed in mental demand (p=0.077, Cohen’s d=0.76) and physical demand (p=0.157, Cohen’s d=0.81), indicating that the two paradigms impose comparable levels of cognitive and physical load. In contrast, the 3D-Morph paradigm demonstrates significantly lower scores in temporal demand (p<0.01, Cohen’s d=1.35), performance (p<0.01, Cohen’s d=1.77), effort (p=0.021, Cohen’s d=0.94), and frustration (p<0.01, Cohen’s d=1.60). These findings suggest that the 3D-Morph paradigm can effectively reduce task completion time, improve operational efficiency and system performance, and alleviate user frustration, thereby enhancing overall usability in P300-based BCI applications.

## 4. Discussion

In this study, we proposed a novel P300 stimulation paradigm in which conventional 2D button flickering was replaced by 3D morphological transformations of realistic virtual objects. By inducing P300 responses through dynamic transitions from planar shapes to 3D forms, the proposed approach eliminated the reliance on color-based flickering while preserving the immersive characteristics of the virtual environment. To evaluate the effectiveness of this paradigm, we conducted a systematic comparison between the proposed 3D-Morph paradigm and a traditional 2D paradigm in terms of ACC, ITR and RT. The experimental results demonstrated that the 3D-Morph paradigm not only maintained a favorable user experience but also achieved superior performance. These findings suggest that morphology-based 3D stimulation constitutes a viable and effective design strategy for immersive P300-based BCI systems.

### 4.1. Effects of 2D/3D-Morph Paradigm

Recent studies have demonstrated that the characteristics of visual stimulation play a critical role in modulating ERP amplitudes, particularly for the P300 component, where 3D visual stimuli generally elicit stronger responses than conventional 2D stimuli [45,46,47]. A similar trend was observed in this study. As illustrated in Figure 7, within the canonical P300 time window (200–400 ms) [48], the proposed 3D-Morph paradigm consistently elicited higher P300 amplitudes than the 2D paradigm across all eight analyzed electrode channels. More specifically, channels located in the frontal (F3, F4) and central (C3, C4) regions exhibited relatively stable P300 responses under both paradigms, with only moderate amplitude increases observed in the 3D-Morph condition. In contrast, substantially larger differences were observed in the parietal (P3, P4) and occipital (O1, O2) regions. For instance, under the 3D-Morph paradigm, the P300 amplitude at channel O1 increased from 1.6776 μV to 3.3326 μV, approximately doubling in magnitude. Similar enhancements were observed at P4 (1.4684 μV to 3.0071 μV), P3 (0.8154 μV to 2.6694 μV), and O2 (0.4159 μV to 3.1776 μV). These results indicate that the proposed paradigm is particularly effective in enhancing ERP responses in parietal and occipital regions, which are closely associated with visual processing. In general, increased ERP amplitudes are positively correlated with improved ACC [26]. This relationship was further supported by both offline and online experimental results. As shown in Figure 6, from the fourth round onward, the 3D-Morph paradigm achieved significantly higher ACC and ITR than the 2D paradigm (both p<0.05), and the performance gap continued to widen as the number of rounds increased (8–10 rounds, p<0.01). At the tenth round, the difference in ACC reached its maximum, increasing from 87.29% (2D) to 94.17% (3D-Morph), corresponding to an improvement of 6.88% (Figure 6a). Consistent trends were observed in the online experiments. The 3D-Morph paradigm (Table 3) achieved an average ACC of 92.50%, compared to 84.58% for the 2D paradigm (Table 4). Meanwhile, the average ITR increased from 28.74 bits/min to 37.23 bits/min, accompanied by a reduction in RT of approximately 0.39 s. These findings suggest that, under identical stimulus conditions, the 3D-Morph paradigm provides superior system performance compared with the conventional 2D paradigm, with its advantages becoming more pronounced at higher numbers of stimulus rounds.

### 4.2. Effects of Adaptive Stopping on System Performance

Conventional fixed-round designs in P300-based BCI systems inherently suffer from a fundamental trade-off between ACC and ITR. Employing a larger number of stimulus rounds (e.g., 10 rounds) can ensure reliable ACC; however, it inevitably prolongs the RT, thereby reducing the overall ITR. Conversely, reducing the number of rounds shortens RT but often leads to degraded ACC due to the inherently low signal-to-noise ratio of EEG signals, thus compromising interaction reliability. In contrast, adaptive or dynamic stopping mechanisms provide a more flexible solution by reducing redundant stimulus repetitions while maintaining ACC [40]. In addition to ACC, ITR, and RT, the processing time per stimulation round (PT) is also an important factor for evaluating online BCI systems, because excessive computational overhead may delay real-time decision-making and weaken the practical advantage of adaptive stopping. Therefore, an effective adaptive stopping algorithm should not only reduce unnecessary stimulus repetitions, but also maintain low per-round processing latency.

In this study, the proposed SA-BLDA algorithm effectively balances this trade-off by adaptively adjusting the number of stimulus rounds. As shown in the offline analysis in Figure 6a, when the number of rounds was fewer than four, both the 2D and 3D-Morph paradigms yielded relatively low ACC (both <70%), with no statistically significant difference between them (p>0.05, Cohen’s d=0.22), indicating that the elicited ERP responses were insufficient for reliable classification. As the number of rounds increased to 4 to 7, statistically significant improvements in both ACC and ITR were observed for both paradigms. However, when the number of rounds further increased to 8 to 10, although ACC continued to improve, no significant difference in ITR was observed, suggesting diminishing returns in ITR beyond seven rounds. Based on these observations, an adaptive range of 4–7 rounds was selected for the online experiments.

The effectiveness of the adaptive stopping strategy was further validated by the online results. As reported in Table 3, under the 3D-Morph paradigm, the SA-BLDA algorithm determined an average of 4.76±0.37 stimulus rounds. Under comparable conditions, the online ACC (92.50%) was substantially higher than that of the offline result at the fifth round (82.08%). Although the online ACC (approximately five rounds) was slightly lower than the best offline performance achieved at the tenth round (94.17%), the ITR was significantly improved (37.23 bits/min vs. 25.50 bits/min). More importantly, SA-BLDA achieved this adaptive decision process with a low computational cost. The PT under the 3D-Morph paradigm was only 26.40±9.41 ms per stimulation round, indicating that the confidence-based stopping decision could be completed rapidly during online operation without introducing substantial additional latency.

A similar trend was observed for the 2D paradigm. As reported in Table 4, the average number of adaptive rounds in the online experiment was 5.30±0.64, with an ACC (84.58%) higher than that of the offline sixth-round condition (75.83%). Although slightly lower than the best offline ACC at the tenth round (87.29%), the online experiment achieved a higher ITR (28.74 bits/min vs. 22.75 bits/min). On the order hand, the PT of SA-BLDA in the 2D paradigm was 48.54±10.47 ms per stimulation round, which was significantly higher than that in the 3D-Morph paradigm (26.40±9.41 ms; p<0.01, Cohen’s d=2.34). This corresponds to a 45.61% reduction in per-round processing time under the 3D-Morph paradigm. Considering both the reduced number of stimulation rounds and the shorter PT, the total computational cost required for online command recognition was further decreased, which is beneficial for maintaining system responsiveness in real-time BCI applications.

### 4.3. Workload Evaluation Analysis

In practical P300-based BCI applications, prolonged exposure to repetitive high-round visual stimulation can induce fatigue, which in turn degrades system performance [49]. To evaluate the impact of the proposed 3D-Morph paradigm and the SA-BLDA adaptive stopping strategy on subjective workload, the NASA Task Load Index (NASA-TLX) was administered to 24 participants following the experiments under both 2D and 3D-Morph conditions.

The results indicate that the mental demand (23.75) and physical demand (14.79) scores under the 3D-Morph paradigm were not significantly different from those under the 2D paradigm (27.29 and 17.71, respectively; p=0.077 and p=0.157), suggesting that the proposed paradigm does not impose additional cognitive or physical burden on participants. As reported in Table 3, the average number of stimulus rounds under the 3D-Morph paradigm was reduced to 4.76 ± 0.37, compared with 5.30 ± 0.64 for the 2D paradigm (Table 4). Correspondingly, the average RT decreased from 6.33 ± 0.52 s to 5.87 ± 0.49 s, resulting in a significant reduction in temporal demand (3D-Morph: 17.92 vs. 2D: 28.13, p<0.01), indicating that participants were able to complete target recognition tasks more efficiently. Significant improvements were also observed in performance and effort scores. Specifically, the performance score was significantly lower (better) under the 3D-Morph paradigm (17.08 vs. 27.50, p<0.01), and the effort score was also reduced (18.75 vs. 21.88, p=0.036), suggesting that participants achieved better task outcomes with less perceived effort. In addition, the frustration score under the 3D-Morph paradigm (16.25) was significantly lower than that of the 2D paradigm (27.29, p<0.01), indicating a reduction in task-related frustration, likely due to improved target recognition reliability. Furthermore, no participants reported symptoms of cybersickness, which can be attributed to the simple and intuitive interface design and the smooth morphological transitions of the 3D-Morph paradigm.

Overall, the NASA-TLX results suggest that integrating the 3D-Morph paradigm with the SA-BLDA adaptive stopping strategy effectively alleviates subjective workload, facilitating the long-term usability of P300-BCI systems in real-world scenarios.

### 4.4. Analysis of Individual Differences

**Figure 9:**
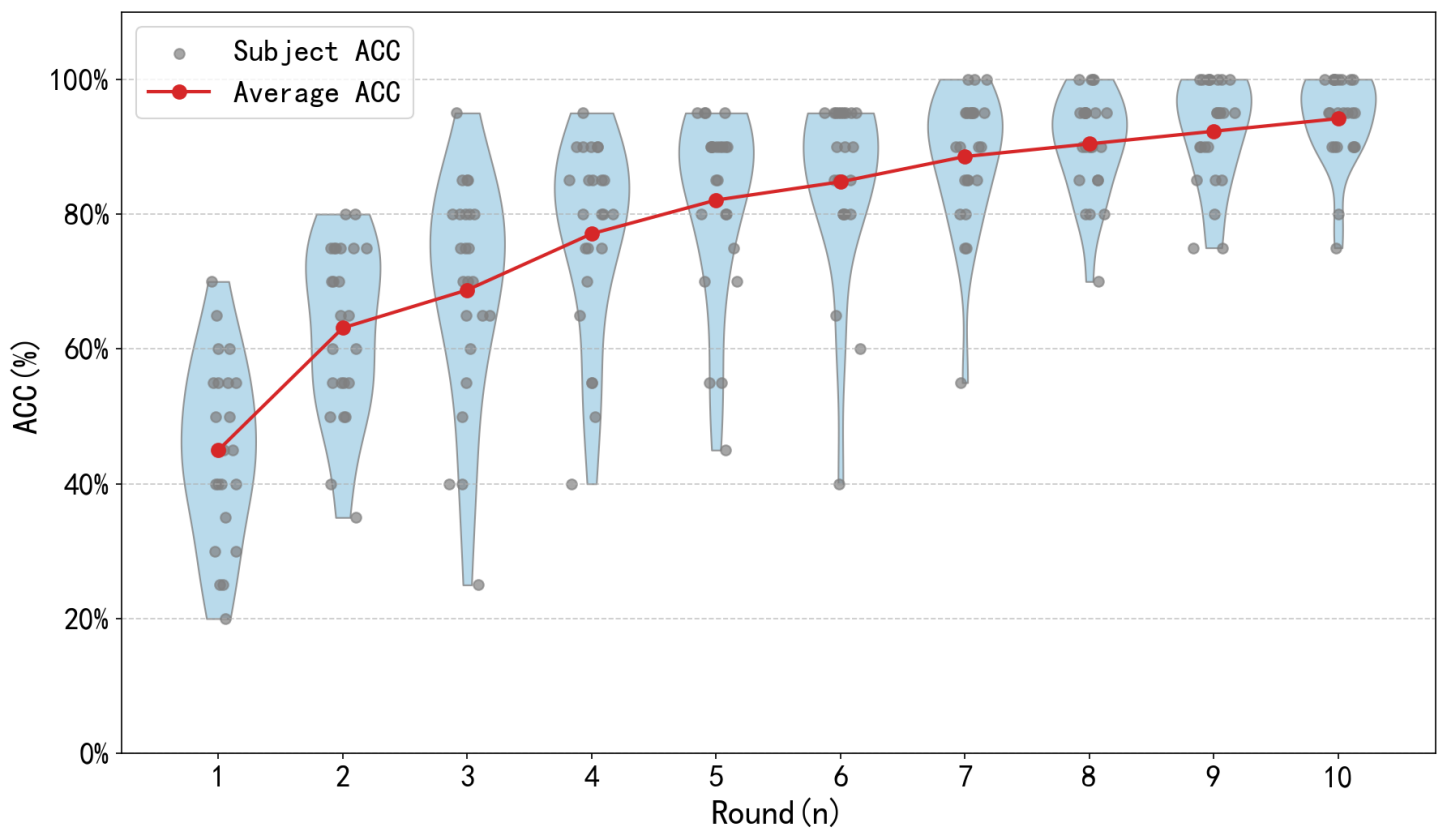
Distribution and mean trend of target ACC across 10 consecutive rounds for 24 subjects in the 3D-Morph Paradigm.

We analyzed the round-wise offline accuracy across all subjects to visualize the subject-level performance variation as the number of stimulation rounds increased. As shown in Figure 9, the round-wise offline ACC increased progressively with the number of stimulation rounds. Each scatter point represents the ACC of an individual subject in a given round and the red line indicates the mean ACC across all 24 subjects. However, the scatter distribution and boxplots also revealed clear inter-subject variability. In the early rounds, the ACC values were widely distributed across subjects, indicating that some subjects could achieve relatively stable recognition with fewer stimulation rounds, whereas others required more repetitions. As the number of rounds increased, the distribution became more concentrated and the average ACC improved, suggesting that additional stimulus accumulation reduced, but did not completely eliminate, individual differences.

**Figure 10:**
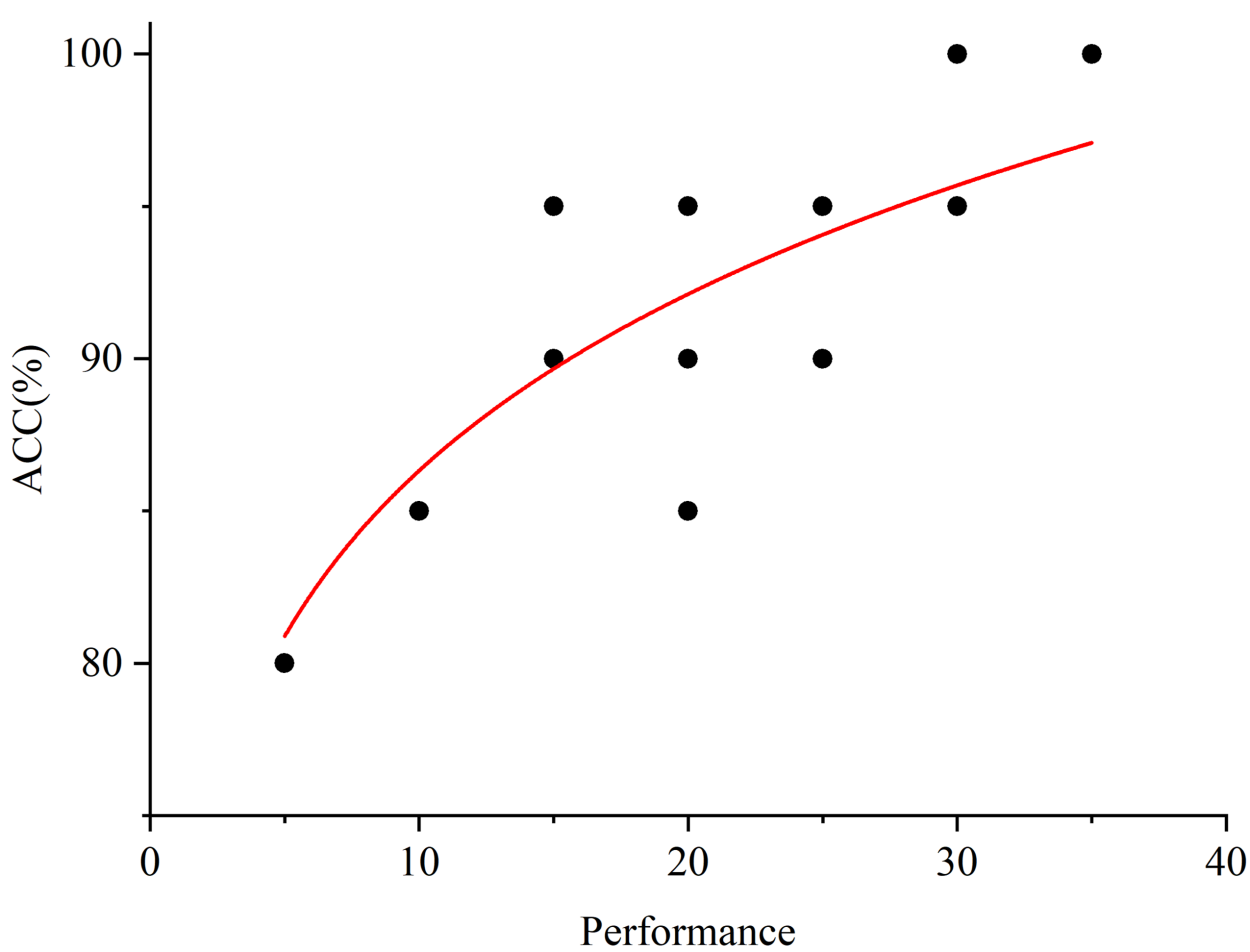
Correlation between online ACC and NASA-TLX performance subscale score across subjects. Black dots represent individual subjects, where each pointindicates a subject’s online ACC and corresponding NASA-TLX performance subscale score. The red line denotes the fitted trend line.

In addition, to further examine the individual differences observed in the online experiment, we analyzed the association between objective online performance and subjective self-rated performance (Figure 10). Specifically, the online ACC of each subject was correlated with the NASA-TLX performance subscale score. A strong positive correlation was observed between online ACC and the NASA-TLX performance subscale score across subjects, with a correlation coefficient of r = 0.98. This finding provides evidence that the inter-subject variability in online BCI control was not merely random fluctuation in the decoding results, but was also reflected in the subjects’ subjective experience. Subjects with more stable online recognition performance may have perceived stronger control over the system, resulting in higher self-rated performance.

### 4.5. Limitations and Future Work

Although the proposed 3D-Morph paradigm combined with the SA-BLDA strategy showed promising performance, several limitations should be acknowledged.

First, the present study was limited to 24 healthy young participants aged 20–25 years, restricting the generalizability of the findings. Future work should recruit larger and more diverse populations, including clinical users with motor impairments, and validate the system in real-world assistive-control tasks using practical outcomes such as task completion time, error recovery, workload, safety, and user acceptance.

Second, the experiment was conducted at a single site with a fixed VR-BCI hardware configuration. Moreover, visual fatigue, engagement, comfort, cybersickness, and workload were not quantified using standardized questionnaires or objective indicators. Future studies should combine subjective scales with eye-tracking, blink rate, fixation patterns, pupil diameter, behavioral responses, and physiological measures to assess usability more comprehensively.

Third, the present study only evaluated short-term performance. Long-term stability, learning effects, user adaptation, and fatigue accumulation across repeated sessions remain unclear. Future multi-session studies are needed to determine whether the proposed 3D-Morph paradigm and SA-BLDA strategy can maintain stable performance over time.

Fourth, the offline and online evaluations were not based on strictly equivalent decision rules. The offline experiment used fixed 10-round stimulation to estimate the performance upper bound, whereas the online experiment used SA-BLDA to adaptively stop within 3 to 7 rounds. Therefore, the offline 10th-round result reflects maximum information accumulation, while the online result represents a trade-off between accuracy and interaction efficiency. Future studies should include fixed-round online controls, such as 5- or 7-round conditions, to enable fairer comparisons.

Finally, the current interface still followed a conventional 2 × 4 matrix layout. Although this design preserved comparability with classical P300 interfaces, it did not fully exploit the spatial affordances of immersive VR. Different 3D layouts, such as depth-layered, non-coplanar, circular, or surrounding arrangements, may affect visual search, attention allocation, P300 elicitation, and user experience [27,50]. Future studies should systematically compare these layouts to establish design principles for immersive P300-BCI interfaces.

## 5. Conclusions

In this study, an immersive P300-based BCI system integrating the 3D-Morph stimulation paradigm and the SA-BLDA adaptive classification strategy was proposed. By replacing conventional color-flashing stimuli with 2D-to-3D morphological transformations, the proposed paradigm was designed to provide a more visually dynamic stimulation form and to elicit target-related ERP responses under the tested P300-BCI setting. In addition, the SA-BLDA strategy adaptively adjusts the number of stimulus rounds based on classification confidence, which was associated with favorable ACC and ITR values in the present experiments.

Experimental results showed that, at the group level, the proposed 3D-Morph paradigm achieved higher average ACC and ITR than the conventional 2D paradigm in both offline and online evaluations. In the offline experiments, the 3D-Morph paradigm achieved an average ACC of 94.17% and an ITR of 25.50 bits/min, compared with 87.29% and 22.75 bits/min for the 2D paradigm, respectively. In the online experiments, the 3D-Morph paradigm achieved an average ACC of 91.46% and an ITR of 37.23 bits/min, corresponding to an ACC increase of 7.50 percentage points and an ITR improvement of 29.54% compared with the 2D paradigm. Moreover, the average RT was reduced from 6.33 s to 5.87 s, suggesting improved response speed under the current online task setting. In addition, the processing time per stimulation round for the SA-BLDA algorithm was substantially reduced from 48.54 ms in the 2D paradigm to 26.40 ms in the 3D-Morph paradigm, representing a 45.61% reduction. This result indicates that the proposed paradigm not only improved recognition performance but also reduced the computational time required for online decision making, thereby supporting more efficient real-time BCI operation. Furthermore, subjective workload evaluation using NASA-TLX revealed that the 3D-Morph paradigm was associated with lower subjective temporal demand, perceived effort, and frustration, as well as better self-rated performance, compared with the 2D paradigm. These findings suggest that the proposed approach may improve selected BCI performance metrics, enhance online processing efficiency, and reduce perceived workload under the present experimental conditions.

Overall, the integration of the 3D-Morph stimulation paradigm and the SA-BLDA adaptive strategy represents a feasible approach for improving selected efficiency-related metrics of P300-based BCI systems, while potentially reducing subjective workload. However, the generalizability of these findings remains to be further validated. Future studies with larger cohorts, more diverse user groups, longitudinal evaluations, and application-oriented tasks are needed to determine the robustness and practical utility of the proposed approach.

## Figures and Tables

**Table 1:** Definitions of parameters used in Figure 4 and Algorithm 1.

Parameter	Definition
$L_{i}$	Current number of stimulation rounds at the *i*-th update.
$L_{\min}$	Minimum number of stimulation rounds required before a valid decision can be made.
$L_{\max}$	Maximum number of stimulation rounds allowed for button recognition.
$\theta_{0}$	Predefined control-output threshold for adaptive stopping.
Δθ	Confidence difference used to determine whether additional stimulation rounds are required.
*u*	Regression score vector obtained from the BLDA classifier.
$u_{\max}$	Maximum score in the regression score vector *u*, corresponding to the most likely target.
$u_{\sec}$	Second-largest score in the regression score vector *u*.
$f_{i}$	Feature vector obtained at the *i*-th update.
$F_{i}$	Feature matrix constructed from the accumulated feature vectors at the *i*-th update.
$L_{i+1}$	Updated number of stimulation rounds for the next iteration.
$F_{i+1}$	Updated feature matrix for the next iteration.

**Table 2:** Key parameters used in the SA-BLDA algorithm.

Parameter	Value
$\theta_{0}$	0.2
Δθ	Actual value used in the algorithm
$L_{max}$	7
$L_{min}$	3
$u_{max}$	Computed online
$u_{sec}$	Computed online

**Table 3:** Individual online experimental results and standard deviations of 24 participants under the 3D-Morph paradigm.

Subject	Avg. Round	RT (s)	ITR (bits/min)	ACC (%)	PT (ms)
S1	5.20	6.24	32.98	90.00	34.12
S2	5.05	6.07	30.39	85.00	31.08
S3	5.25	6.46	35.50	95.00	40.35
S4	4.50	5.65	36.39	90.00	21.76
S5	3.95	5.10	44.95	95.00	10.24
S6	4.05	5.35	48.49	100.00	14.63
S7	4.95	5.91	43.88	100.00	27.91
S8	4.95	6.44	35.60	95.00	39.47
S9	4.75	5.67	36.27	90.00	22.59
S10	5.20	6.24	29.57	85.00	33.21
S11	4.50	5.83	39.32	95.00	25.37
S12	4.60	5.45	37.71	90.00	16.82
S13	4.65	5.52	41.59	95.00	18.55
S14	4.85	5.79	35.50	90.00	24.73
S15	4.60	5.72	28.82	80.00	23.69
S16	4.95	5.94	38.62	95.00	28.84
S17	4.75	5.73	35.89	90.00	23.18
S18	4.95	5.77	39.77	95.00	24.96
S19	4.70	5.63	32.75	85.00	20.41
S20	4.60	5.26	43.58	95.00	12.35
S21	5.10	6.93	26.62	85.00	49.72
S22	4.40	5.70	36.05	90.00	22.07
S23	4.70	6.05	37.87	95.00	30.66
S24	5.05	6.32	32.52	90.00	36.89
Average ± STD	4.76 ± 0.37	5.87 ± 0.49	37.23 ± 5.20	91.46 ± 5.58	26.40 ± 9.41

**Table 4:** Individual online experimental results and standard deviations of 24 participants under the 2D paradigm.

Subject	Avg. Round	RT (s)	ITR (bits/min)	ACC (%)	PT (ms)
S1	6.85	7.29	26.23	80.00	65.31
S2	5.80	7.24	25.46	85.00	64.18
S3	6.50	6.54	28.19	85.00	55.63
S4	4.90	6.13	26.94	80.00	44.86
S5	4.85	5.96	34.51	90.00	41.28
S6	4.70	6.14	30.03	85.00	45.19
S7	5.05	6.51	25.36	80.00	54.82
S8	4.45	5.74	28.75	80.00	37.46
S9	5.05	6.51	31.58	90.00	54.15
S10	5.85	6.28	26.27	80.00	48.27
S11	5.25	6.93	23.80	80.00	59.47
S12	4.80	6.11	33.69	90.00	43.54
S13	4.85	5.71	36.04	90.00	35.54
S14	4.40	5.55	41.31	95.00	32.15
S15	4.95	5.91	24.87	75.00	40.15
S16	6.60	7.05	33.97	90.00	61.25
S17	5.70	6.25	32.67	85.00	47.52
S18	4.85	5.70	25.80	75.00	34.82
S19	5.15	6.46	28.55	85.00	52.79
S20	4.75	5.74	32.15	85.00	36.83
S21	5.75	7.50	19.59	75.00	68.72
S22	4.70	5.81	31.75	85.00	38.72
S23	5.35	6.43	28.67	85.00	51.46
S24	6.00	6.41	28.79	85.00	50.83
Average ± STD	5.30 ± 0.64	6.33 ± 0.52	28.74 ± 3.92	83.96 ± 5.47	48.54 ± 10.47

## Data Availability

The data sets presented in this study are available on request from the corresponding authors.
